# Tailoring the Textural Characteristics of Fat-Free Fermented Concentrated Milk-Protein Based Microgel Dispersions by Way of Upstream, Downstream and Post-Production Thermal Inputs

**DOI:** 10.3390/foods11050635

**Published:** 2022-02-22

**Authors:** Anisa Heck, Stefan Nöbel, Bernd Hitzmann, Jörg Hinrichs

**Affiliations:** Institute of Food Science and Biotechnology, University of Hohenheim, D-70593 Stuttgart, Germany; stefan.noebel@mri.bund.de (S.N.); bernd.hitzmann@uni-hohenheim.de (B.H.); j.hinrichs@uni-hohenheim.de (J.H.)

**Keywords:** fresh cheese, fermented milk gel, texture, process-control, high-protein, dairy

## Abstract

There is a growing demand for new strategies to tailor the texture of fat-free fermented concentrated milk products, also referred to as milk protein-based (MPb) microgel dispersions. Methods should be easy to incorporate into the production scheme, offer labelling without added components and be cost-efficient. Thermal treatments are traditionally used upstream (milk heating) and downstream (pre-concentration heating) in the production of these dispersions, though there is little knowledge as to the effects that combinations of different thermal input levels have on final texture. Therefore, this study investigated combinations of thermal input at different intensities and steps in the production scheme at the pilot scale and the relationships with texture. We demonstrated that increasing the intensity of upstream milk heat treatment, in combination with downstream pre-concentration heating, increases gel firmness and apparent viscosity. Downstream pre-concentration heating produces final fat-free fermented concentrated MPb microgel particles that are resistant to post-heating aggregation. On the other hand, omission of downstream pre-concentration heating results in smaller particles that are sensitive to post-heating aggregation. Furthermore, gel firmness and apparent viscosity increase with post-heating. Consequently, combining different levels of thermal inputs upstream, downstream (pre-concentration) and post-production, can produce fat-free fermented concentrated MPb microgel dispersions with a range of different textures.

## 1. Introduction

A number of fat-free fermented concentrated milk products, such fat-free fresh cheese, quark, and skyr, have similar structures consisting of protein aggregates containing a large amount of solvent [1]. Because of these characteristics, they can be classified as dispersions of milk-protein based (MPb) microgel particles. The amount of solvent in the protein aggregate network changes in response to environmental and solvent conditions, which results in swelling or shrinkage of the microgel particles [1,2]. In concentrated dispersions, microgel particles are closely packed and rotation is impeded (*ϕ* > 0.4) [1]. At this point, the microgel particles are elastically deformed or break up when too high forces are acting upon them. It follows that the macrostructural behavior of microgel dispersions, more specifically, for concentrated dispersions, is defined by the microgel particle structure [3].

In the production of fat-free fermented concentrated MPb microgel dispersions, the fermentation of skim milk results in the development of a milk protein- (casein)-based gel [4,5,6]. The gel is broken into small (gel) particles and concentrated. Ideally, the resulting product is smooth, soft, uniform, free from lumps and without whey separation. In spite of this, undesirable lumpiness or graininess can occur in fat-free fermented MPb microgel dispersions, e.g., as shown for yoghurt and fresh cheese [7,8,9]. In addition to this, the desire to create innovative products with tailored textures gives rise to the demand for strategies to transform the texture of fat-free fermented milk products. A number of strategies have been investigated, including mechanical treatment [10,11], exopolysaccharide-producing cultures [9,12,13], and adding components such as whey proteins [14]. However, there are often drawbacks to employing a particular strategy, such as increased syneresis, undesired textural changes like increased ropiness, and additional labelling requirements.

Thermal input has long been used to alter the textural characteristics of fat-free fermented concentrated MPb microgel dispersions. The state of the literature regarding the texture of fat-free fermented concentrated MPb microgel dispersions and the relationships with processing parameters, including thermal input at various process steps, was recently reviewed [3]; we summarize the relevant points here. There is a large amount of information available regarding upstream thermal treatments of milk and the impacts on the textural properties of fat-free fermented concentrated MPb microgel dispersions [5,15,16]. Fat-free fermented concentrated MPb microgel dispersions produced using skim milk heated at pasteurization conditions, e.g., 72 °C for 15 s, are softer and have a lower viscosity, since whey proteins remain in their native states and cause the formation of large microgel particles with large pores [16]. By heating the milk at 82–95 °C for 3–6 min, 75 to 90% whey protein denaturation is achieved, resulting in a firm and creamy texture, and high yield [16,17]. A combination of upstream thermal input and downstream pre-concentration heating, e.g., the *Westfalia-thermo* process, is preferred, in order to optimize protein yield and improve shelf-life [18,19,20]. This process includes upstream heat treatment at 95–96 °C for 2–3 min and downstream pre-concentration heating at 62–80 °C for 3–20 min [3]. Despite widespread application in technical scale production of fat-free fermented concentrated MPb microgel dispersions, very little is known about the relationship between texture and pre-concentration heating. Post-heating of the final concentrated product has been proposed as a method to increase rheological properties, where post-heating at 45 and 54 °C for 300 min significantly increased gel firmness and apparent viscosity of fat-free fermented concentrated MPb microgel dispersions compared to post-heating at 38 °C (300 min) [21]. However, particle size also increased to greater than the threshold for sensory perception of graininess (d_75,3_ > 40 um) with post-heating at 45 and 54 °C, demonstrating a major drawback of post-heating for altering texture [22]. In a recent publication, we demonstrated that production of fat-free fermented concentrated MPb microgel dispersions with combined steps of pre-concentration heating and post-heating at 38 °C does not lead to a significant increase in particle size, whereas particle sizes increase in samples produced without pre-concentration heating followed by post-heating at the same temperature [23]. The thermal treatment of milk and multiple levels of post-heating for samples with and without pre-concentration heating were not investigated in this previous study. Despite knowledge that thermal input significantly alters the textural characteristics of fat-free fermented concentrated MPb microgel dispersions, to the best of our knowledge, no studies exist that explore the impact of combinations, and intensities therefore, of thermal input at multiple stages in the production scheme.

The current study aimed to modify the texture of a fat-free fermented concentrated MPb microgel dispersion, namely, fresh cheese, by combining upstream, downstream and post-production thermal inputs. Three thermal processing steps were varied in the production scheme for fresh cheese. First, the intensity of milk heat treatment was varied upstream. Second, heating of the fat-free fermented MPb microgel dispersion was included or omitted before concentration (downstream). Finally, the concentrated dispersion was tempered at different temperatures (post-heating).

## 2. Materials and Methods

### 2.1. Fat-Free Fermented Concentrated Milk Protein-Based Microgel Dispersions

**Figure 1:**
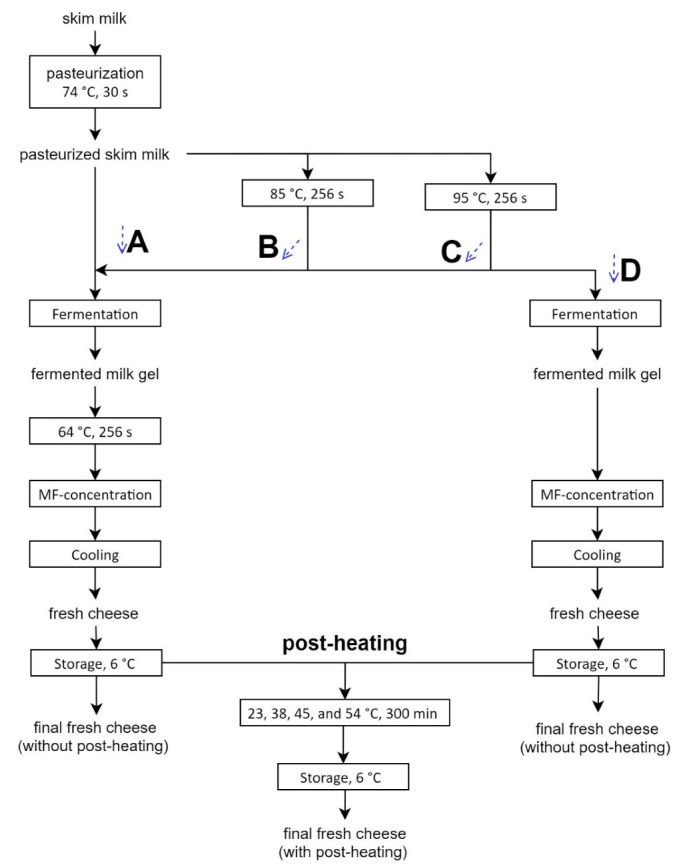
Flow chart depicting the processing parameters for fat-free fermented concentrated milk protein-based microgel dispersions, process variants A, B, C and D.

Fresh raw milk (Meiereihof, University of Hohenheim) was separated (<0.1% *w*/*w* fat) and pasteurized in-house (74 °C for 30 s). The pasteurized milk was used to produce fat-free fermented concentrated milk protein-based microgel dispersions, namely, fresh cheeses, as described by Heck et al. [23]. Modifications of upstream heat treatment (milk), downstream heat treatment (pre-concentration) and post-heating (post-production) were conducted as illustrated in Figure 1.

Different combinations of upstream and downstream treatments are labelled as process variants A, B, C and D. Differing inputs at the post-heating step are indicated by the temperature that was employed or referred to as being without post-treatment (storage at 6 °C). In brief, the milk for process variant A had no additional heat treatment after pasteurization. The milk for process variant B was heated at 80 °C for 256 s and for process variants C and D, the milks were heated at 95 °C for 256 s, leading to approximately 70 and ≥90% denaturation of whey proteins, respectively [24]. Fermentation was carried out by suspending 0.04% *w*/*w* CHOOZIT 230 (Danisco, Niebüll, Germany) mesophilic starter culture containing *Lactococcus lactis* subsp. *lactis* and *cremoris,* and adding 1 mL 100/L rennet (ChyMax Plus; Chr. Hansen GmbH, Nienburg, Germany; min. 190 IMCU/mL) to the heated skim milk. Fermentation was conducted at 22.5 °C to a final pH of 4.45 to 4.50. Following fermentation, the gels were broken up, and for process variants A, B and C, were heated (tubular plant, 150 L/h, ASEPTO-Therm; Asepto GmbH, Dinkelscherben, Germany) to 64 °C for 256 s min and cooled to 38 °C; this step was omitted for process variant D. All process variants were concentrated by microfiltration (MF, 38 °C, nominal pore size 0.06 μm) to final protein contents of 8.2–9.9% *w*/*w*. The fat-free fermented concentrated MPb microgel dispersions for all process variants were cooled to ≤14 °C (ΔT = 24 K in <10 s) by pumping the fermented concentrated milk gel through a screw pump (250–255 L/h, Nemo nM021; Netzsch Mohnopumpen GmbH, Waldkraiburg, Germany) attached to a double-walled heat exchanger cooled with ice water (length 1.86 m, active area 0.4 m^2^). The cooled dispersions were filled directly into 100 mL glass jars and stored at 6 °C. After cold storage for 14–18 h, the glass jars were tempered in a water bath at 23, 38, 45 and 54 °C for 300 min. The glass jars were cooled in an ice bath for 15 min, followed by storage at 6 °C (minimum of 18 h) until further analysis. Each process variant was produced at least three times (i ≥ 3) and the subsequent post-heating was carried out for each of the process variant production replicates.

### 2.2. Particle Size

Particle size was analyzed using static laser light scattering (Beckman Coulter LS 13 320, connected to a Universal Liquid Module and control software v6.01, Beckman Coulter Inc., Miami, FL, USA) according to Heck et al. [25]. The imaginary refractive indices (RI) for particles and water were fixed at 0.00, for white or transparent materials [26]. Since optical properties of microgel properties, such as the RI, are reported to be a function of microgel particle properties, such as swelling and deswelling [27,28], the relationship between the real RI for each sample (i.e., process variant + post-heating treatment) and the calculated particle size distribution is of interest. A method modified from Hayakawa et al. [29] was recently applied to fat-free fermented concentrated MPb microgel dispersions produced at technical scale [25] and was likewise used in the present study. First, real RI values ranging from 1.36 to 1.96 were applied to calculate the particle size distributions. According to Heck et al. [25], with increasing real RI values, the real RI at which the volume of submicron particles (<1 μm) Q_3,sm_ is the smallest (or disappears) in the distribution is identified as the apparent real RI (RI_app_). For each sample, the arithmetic mean, standard error and maximum RI_app_ were calculated from ≥6 independent measurements. When the particle size distribution is calculated using a real RI smaller than RI_app_, the volume distribution is skewed by the appearance of the non-existent submicron particles [25]. When calculated using a real RI above this value, the influence of the change in real RI on the volume distribution of particles is at a minimum. Thus, the maximum RI_app_ for each sample was used for further calculations of the particle size distributions. Particle size analysis was conducted two or more times for each sample. Mean cumulative and volume-based particle size distributions were prepared. Equivalent diameters d_50,3_, d_75,3_ and the distribution span (d_90,3_–d_10,3_) were calculated. Arithmetic mean and standard error values are presented in Section 3.

### 2.3. Rheology

To conduct oscillatory and rotational measurements, a stress-controlled rheometer AR 2000 (minimum torque: 9.1 nNm; TA Instruments, Eschborn, Germany) fitted with a concentric cylinder cup and bob system (stator inner radius: 15.0 mm, rotor outer radius: 14.0 mm) was employed. Before sampling, samples were briefly stirred with a plastic spoon. For each measurement, 16–17 g of the sample were transferred into the cup. The sample was equilibrated at 10 °C for 15 min. All rheological measurements were conducted at a minimum in duplicate.

A combined time sweep and flow curve procedure was conducted as described by Körzendörfer et al. [30], in one continuous measurement procedure. Oscillatory measurements in the linear viscoelastic region (γ = 0.0025) in the form of a 30 s time sweep at a constant angular frequency of 10 rad/s were carried out. The storage modulus and loss tangent (${G^{\prime }}_{10\;rad/s}$ and $\delta_{10\;rad/s}$), as measures of gel firmness and stability (the ratio of energy lost to energy stored), respectively, were determined from this measurement. After a 1 min equilibration period, the shear rate was linearly increased from 0 to 500 1/s within 3 min. This was subsequently followed by a hold step at 500 1/s for 3 min. In the final step, the shear rate was decreased to 0 /s within 3 min in a linear fashion. The apparent viscosity in the upward ramp at a shear rate of 100 1/s ($\eta_{100\;1/s}$) was calculated, chosen to represent oral shear rates [31]. The energy loss (ΔE) was calculated by subtracting the integral of the apparent viscosities over the downward ramp of the shear rate from the upward ramp of the shear rate. This represents the amount of energy required to break down the structure of the sample during the rotational procedure (shear rate: upward ramp, hold step and downward ramp) and is reported in J/m^3^.

The yield stress *σ*_y_ was evaluated using a stress sweep according to Fysun et al. [32]. Using a new sample, also equilibrated at 10 °C for 15 min, the stress was increased from *σ =* 0.05 to 100 Pa (ten points per decade) at a fixed frequency of ω = 1 rad/s. The yield stress *σ*_y_ was taken as the stress at the crossover of storage and loss moduli $G^{\prime }$=$G^{\prime \prime }$.

### 2.4. Chemical Analysis

The pH_20 °C_ was measured using the standard method for milk and milk products (C 8.2, VDLUFA, 2003). The pH_20 °C_ of the heated milk, permeate, fermented milk and fermented concentrated MPb microgel dispersion (without post-heating) were evaluated. Dry matter contents of the fermented concentrated MPb microgel dispersions (without post-heating) were evaluated according to the sea sand method (C 35.3, VDLUFA, 2003). The protein contents of the milk, permeate, and fermented concentrated MPb microgel dispersion (without post-heating) were assessed using the method of Dumas (IDF 185) with a nitrogen analyzer (Dumatherm DT; C. Gerhardt GmbH & Co. KG, Königswinter, Germany). To calculate the protein content, the total nitrogen content was multiplied by a conversion factor of 6.38.

### 2.5. Statistical Analysis

The arithmetic mean values and standard errors for values from multiple batches of fat-free fermented concentrated MPb microgel dispersions (i ≥ 3) were calculated and are presented in Section 3. Significance of the differences between pH, protein and dry matter contents of the process variants, without post-heating were determined by analysis of variance (ANOVA). Analysis of co-variance (ANCOVA) was conducted to evaluate differences between process variants without post-heating, with protein as a covariate. ANCOVA was used to identify significant differences between post-heating temperatures for each process variant. Protein was defined as a covariate and post-heating temperature treatment was defined as a nested factor within the process variant. All tests were complemented by the Tukey-Kramer post-hoc test (α = 0.05) to identify significant differences where appropriate. The software Minitab v19.2020.1 (Minitab Inc., State College, PA, USA) was used to perform all statistical analyses.

## 3. Results and Discussion

The gross composition and physical-chemical characteristics at the different steps of production for process variants A, B, C and D without post-heating are listed in Table 1. There are no significant differences between the pH_20 °C_ values for each process variant after each different process step (heated milk, permeate, fermented milk and fermented concentrated MPb microgel dispersion). Initial milk protein contents are not significantly different between process variants; however, differences are found between protein contents of the permeate (Table 1). Since MF was employed to concentrate the fermented milk gel, proteins not incorporated into the fat-free fermented MPb microgel particles remain in the permeate. Protein and dry matter contents of the fat-free fermented concentrated MPb microgel dispersions for all individual productions range between 8.2–9.9% *w*/*w* and 13.6–14.8% *w*/*w*, respectively; however, are not significantly different between process variants. Furthermore, the individual ranges of protein contents for process variants A, B, C and D are 8.3–9.1, 8.3–9.3, 8.2–9.9 and 8.3–9.1% *w*/*w*, respectively. That being said, protein content is related to rheological properties [3] and must be taken into account with respect to differences between individual productions. Thus, final protein content is considered as a covariate in all further statistical analyses, to account for differences in protein contents between productions of process variants and the relationships with product properties, e.g., apparent viscosity.

### 3.1. Upstream Thermal Input

As shown in Figure 1, the upstream thermal input for process variants A, B and C increases from no additional input (process variant A: pasteurization conditions, 74 °C, 30 s), moderate (process variant B: 74 °C, 30 s + 80 °C, 256 s), to high thermal input (process variant C: 74 °C, 30 s + 95 °C, 256 s). With increasing upstream thermal input, the protein content of the permeate also decreases, where the protein contents of permeates for process variants A, B and C are 1.0 ± 0.2, 0.9 ± 0.2 and 0.6 ± 0.2% *w*/*w*, respectively (Table 1). Though the permeate protein content for process variant A is significantly higher than that for process variant C, that of process variant B is not significantly different from process variants A and B. The intensity of the upstream heat treatment for process variant A (74 °C for 30 s, Figure 1) is not high enough to cause denaturation of whey proteins [16]. In contrast, the intensities of the upstream heat treatments for process variants B (80 °C for 256 s) and C (95 °C for 256 s) are high enough to cause whey protein denaturation. Since denatured whey proteins form complexes with the (casein-based) MPb microgel particles, they are retained in the dispersion once it is concentrated by MF and increase the yield (i.e., decrease protein content of the permeate) [3].

Of the samples without post-heating, the RI_app_ for process variants B and C of 1.45 ± 0.04 and 1.46 ± 0.04, respectively, are significantly higher than for process variant A, with 1.40 ± 0.01 (Table 2). Since the upstream thermal input for both process variants B and C is sufficient for whey protein denaturation, in contrast to the lower thermal input for process variant A, it is hypothesized that the heat-denatured whey proteins incorporated into the MPb microgel particles modify the particle optical properties, resulting in a change in the RI_app_.

**Figure 2:**
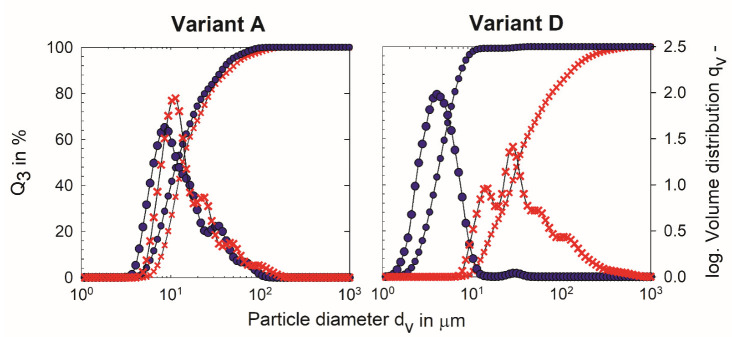
Mean cumulative and volume-based particle size distributions of fat-free fermented concentrated milk protein-based microgel dispersions, process variants A and D without post-heating (6 °C, circles) and with post-heating at 54 °C (crosses) for 300 min (i ≥ 3; *n* ≥ 2).

The cumulative and volume-based particle size distributions, calculated using the maximum RI_app_ for process variant A without post-heating, are displayed in Figure 2 and are representative of the distributions for process variants B and C without post-heating. The main volume of particles in the distributions for process variations A, B and C without post-heating is between 2–20 μm, with a smaller volume of particles ranging up to approximately 100 μm (Figure 2).

There are no significant differences between the d_50,3_, d_75,3_ and the span of process variations A, B and C (without post-heating) (Table 2), despite the different intensities of upstream heat treatment (followed by equal heat treatments pre-concentration, Figure 1). According to Vaziri et al. [16], lower intensities of upstream heat treatments are associated with lower degrees of whey protein denaturation, e.g., heating at 72 °C for 16 s results in large microgel particles, whereas treatments at 82 and 90 °C for 5 min result in smaller microgel particles. No downstream pre-concentration heating was conducted, likely the explanation for the contradictory results compared to the present study.

There is no significant difference between rheological values for process variants A and B (without post-heating), and significant differences between process variant C when compared to process variants A and B, seen for all rheological characteristics in Table 2.

The *σ*_y_ for process variants A and B without post-heating, with values of 0.6 ± 0.4 Pa and 1.1 ± 0.5 Pa, respectively, indicate a low to non-existent yield stress (Table 2). On the contrary, *σ*_y_ of 7.1 ± 1.0 Pa was measured for process variant C without post-heating, which is significantly higher than for process variants A and B (Table 2). The ΔE for process variants A, B and C runs in parallel to the *σ*_y_, where ΔE for process variants A (ΔE = 63 ± 19 J/m^3^) and B (ΔE = 101 ± 30 J/m^3^) are significantly lower than for process variant C (ΔE = 185 ± 22 J/m^3^) without post-heating, though are not significantly different from each other (Table 2).

Both parameters indicate that a higher amount of energy is required to break the structure of process variant C compared to process variants A and B. This stronger structure is due to higher amounts of protein–protein interactions from denatured whey proteins incorporated into the structure, based on a greater upstream thermal input [15]. The level of whey protein denaturation achieved by upstream heat treatment at 80 °C for 256 s (process variant B) was insufficient to achieve a similarly strong structure as that achieved with heating for 256 s at 95 °C. This conclusion is supported by the significantly higher ${G^{\prime }}_{10\;rad/s}\;$ for process variant C (562 ± 51 Pa) compared to process variants A (26 ± 11 Pa) and B (60 ± 10 Pa) (Table 2). The $\delta_{10\;rad/s}$ for process variant C (15.5 ± 0.2) is significantly lower than for process variants A (23.5 ± 2.1) and B (20.1 ± 1.3) (Table 2). A higher $\delta_{10\;rad/s}\;$ value indicates poorer storage of applied mechanical energy, e.g., due to shorter lifetimes of protein–protein bonds [33,34]. This is in line with the values for ΔE, where more energy is required to break down the structure for process variant C when compared to process variants A and B (Table 2). For process variant C, the upstream milk heating at 95 °C for 256 s (combined with downstream pre-concentration heating) likely resulted in high levels of protein denaturation [24] and binding of these proteins with the MPb microgel particles, leading to more stable protein structures and higher gel firmness, making the gel more resistant to shear [3].

**Figure 3:**
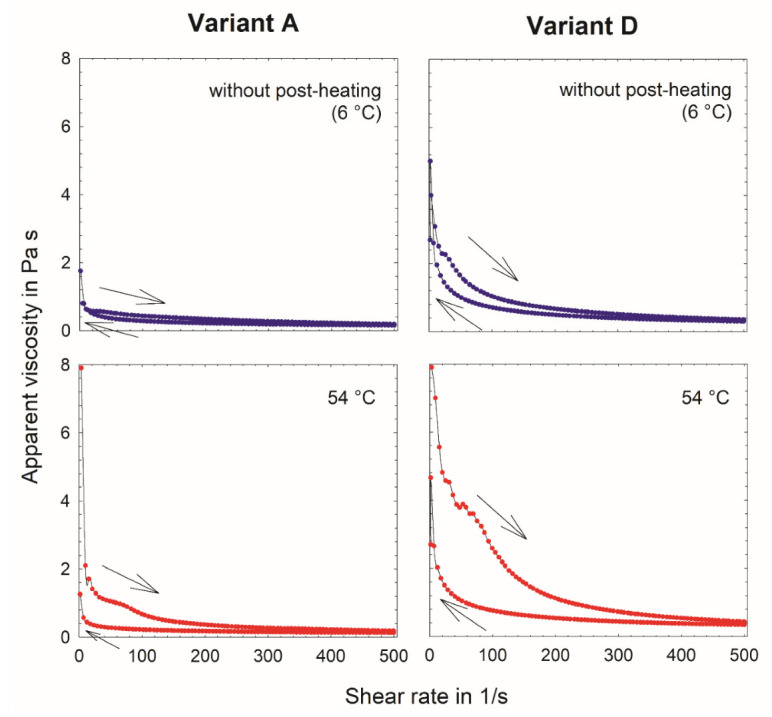
Mean viscosity curves of fat-free fermented concentrated milk protein-based microgel dispersions, process variants A and D without post-heating (6 °C) and with post-heating at 54 °C for 300 min (i ≥ 3; *n* ≥ 2).

The viscosity curve for process variant A without post-heating is depicted in Figure 3. Those for process variants B and C have similar shapes to that of process variant A. The apparent viscosities at a shear rate of 100 1/s ($\eta_{100\;1/s}$) for products A and B without post-heating (process variant A: 0.5 ± 0.1 Pa s; process variant B: 0.7 ± 0.2 Pa s) are significantly lower than for process variant C without post-heating (1.3 ± 0.1 Pa s) (Table 2). This observation is in accordance with the results of ${G^{\prime }}_{10\;rad/s}$ and $\delta_{10\;rad/s},\;$ where only process variant C is significantly different from process variants A and B (without post-heating).

The heat-denatured whey proteins, stemming from the upstream thermal treatment for process variant C, bind serum and take part in gel formation, resulting in higher apparent viscosity and modified flow behavior [3,35]. These results are in line with previous investigations on the impact of upstream heat treatment intensity on the rheological properties of fat-free fermented concentrated MPb microgel dispersions, where apparent viscosity and firmness generally increase along with upstream heat treatment intensity [16,36,37]. Unlike Chever et al. [37], where increases and plateaus of apparent viscosity, firmness and particle size were reported for upstream heating at ≥80 °C compared to 70 °C, no significant increase in apparent viscosity or firmness was achieved at 80 °C in the present study. However, in contrast to the 256 s (4.3 min) heating time in the present experiments, heat treatment was conducted for 7 min in the previous study.

### 3.2. Pre-Concentration Heating

The effect of downstream pre-concentration heating on texture can be examined by comparing process variants with the same thermal input for upstream processing (95 °C for 256 s) and including (process variant C) or omitting pre-concentration heating (process variant D) (Figure 1). Unlike in Section 3.1, where RI_app_ increased along with increasing upstream thermal input, omission of downstream pre-concentration heating is accompanied by a significant increase in RI_app_ (process variant C: 1.46 ± 0.04; process variant D: 1.76 ± 0.03) (Table 2). These results are in line with those from fat-free fermented concentrated MPb microgel dispersions produced at technical scale, where those produced with pre-concentration heating had RI_app_ values between 1.42–1.48 and one product produced without pre-concentration heating had a RI_app_ of 1.74 [25].

A change in size and swelling may impact the optical properties of microgel particles [27,29]. However, a number of main mechanisms have been proposed to explain the change in RI_app_ along with inclusion *versus* omission of pre-concentration heating [25], as follows: The pre-concentration heating step alters the external structure (e.g., by inclusion of additional whey proteins), increases particle voluminosity and causes the internal microgel particle structure to contract, resulting in decreases in the RI_app_ [25]. That being said, there are no significant differences between the protein contents of process variants C and D with regard to the permeate or final dispersion (Table 1). Further investigations should be conducted to clarify the roles of whey protein (and denaturation), voluminosity and internal particle structure on the RI_app._

The cumulative and volume-based particle size distributions for process variant C (without post-heating) are comparable to those of process variant A (without post-heating) (data not shown), and therefore, Figure 2 is used for visual comparisons. The volume-based particle size distribution for process variant D (without post-heating) is wider than that for process variant C (without post-heating), confirmed by the values for span (process variant C: 26.5 ± 1.5 μm; process variant D: 4.9 ± 0.3 μm), which are significantly different (Table 2). For samples without post-heating, the particles in process variant C are generally larger than the particles in process variant D, as confirmed by a comparison of the d_50,3_ and d_75,3_ in Table 2. The d_50,3_ and d_75,3_ are significantly higher for process variant C than for process variant D without post-heating (process variant C: d_50,3_ = 11.9 ± 0.3 μm, d_75,3_ = 19.6 ± 0.6 μm; process variant D d_50,3_ = 4.2 ± 0.2 μm, d_75,3_ = 5.7 ± 0.2 μm).

Parallel to the cumulative and volume-based particle size distributions, the viscosity curve for process variant C (without post-heating) has a similar shape (not shown) to that of process variant A in Figure 3. Though the apparent viscosities are slightly higher, the curve displays the same absence of a distinct yielding behavior. The *σ*_y_ and ΔE for process variant D (*σ*_y_ = 3.9 ± 0.7 Pa and ΔE = 107 ± 26 J/m^3^) are significantly lower than for process variant C (*σ*_y_ = 7.1 ± 1.0 Pa and ΔE = 185 ± 22 J/m^3^) without post-heating (Table 2).

In combination with upstream heating of milk at temperatures high enough to cause whey protein denaturation, downstream pre-concentration heating is said to incorporate higher amounts of whey protein into the gel structure [18]. The theory is that whey proteins that are not denatured during upstream heating undergo conformational changes when the pH is reduced past their isoelectric point. These whey proteins bind to caseins as the pH decreases down to pH 4.5–4.6. This binding is unstable; however, pre-concentration heating (after fermentation) causes caseins to contract and enclose these deposited whey proteins [18].

Despite no significant change in protein content (Table 1), including this pre-concentration heating step (in combination with upstream heating, Figure 1) altered the texture of the fat-free fermented concentrated MPb microgel dispersions in this study. It should be noted that while changes in protein content could have occurred, the method employed to determine protein contents has a low sensitivity, particularly for low protein concentrations, such as those under review. This is in line with previous observations from our lab, where the particle size and volume fraction significantly increased with the inclusion of pre-concentration heating [23]. In contrast to our previous work, we observed a significantly higher apparent viscosity for the process variant with pre-concentration heating. The non-significant difference in the previous study was likely due to a low number of batch replications (i = 1) in comparison to the current experiments (i ≥ 3). The higher voluminosity of MPb microgel particles in dispersions produced with pre-concentration heating provides the basis for higher apparent viscosity, gel firmness, and yield stress [23].

### 3.3. Post-Heating

All process variants (differing upstream thermal input; with and without downstream pre-concentration heating) were treated with an additional thermal step at the final protein concentration, as shown in Figure 1. Therefore, the (i) combinations between upstream thermal input, (ii) thermal input before concentration, and (iii) post-heating temperatures and the effects on sample properties are discussed in this section. From Table 2, the most obvious difference due to post-heating is observed when post-heated samples of process variants with a pre-concentration heating step (process variants A, B and C) are compared to the process variant without pre-concentration heating (process variant D). For example, while there is no significant difference in span and d_75,3_ for post-heating temperatures of up to 54 °C for process variants A and B and up to 45 °C for process variant C, the span of process variant D increases significantly at post-heating temperatures of 38, 45 and 54 °C compared to without post-heating and post-heating at 23 °C (Table 2). This is depicted in Figure 2, where the cumulative and volume-based particle size distributions for process variant A appear very similar without post-heating compared to heating at 54 °C. In contrast, the particle size distribution for process variant D is wider and shifts to larger particle sizes with post-heating (Table 2 & Figure 2).

Hahn et al. [21] reported an increase in particle size with post-heating conditions of 45 and 54 °C for 300 min, where the fat-free fermented concentrated MPb microgel dispersions were produced without pre-concentration heating. According to our previous work [23], pre-concentration heating reduces the aggregation potential of microgel particles in fat-free fermented concentrated MPb microgel dispersions subjected to post-heating, which is confirmed in the present study. Furthermore, we have also shown that this reduced aggregation potential is retained, even with reduced upstream thermal inputs as low as 72 °C for 30 s (Figure 1 & Table 2).

As stated in Section 3.2, pre-concentration heating is said to cause caseins to contract and enclose deposited whey proteins within their structure [18]. It is postulated that this step is essential to reduce the aggregation potential of fat-free fermented concentrated MPb microgel particles subjected to post-heating. When not subjected to pre-concentration heating, the whey proteins that bind to caseins due to acidification [18] are loosely bound at the surface of the casein aggregates. These provide binding points that initiate post-heating aggregation of the fat-free fermented concentrated MPb microgel particles.

Despite minimal changes to the particle size distributions of post-heated samples for process variants with pre-concentration heating (A, B and C), post-heating causes significant changes to the RI_app_ and rheological properties (Table 2). The RI_app_ for process variant A decreases significantly when the post-heating temperature is increased to 45 °C (as compared to 6, 23 and 38 °C), whereas a further temperature increase to 54 °C is not accompanied by a further decrease in RI_app_ (Table 2). For process variants B and C, the RI_app_ decreases significantly at a post-heating temperature of 38 °C (compared to 6 and 23 °C) and does not decrease further with additional increases in post-heating temperatures (Table 2). Similar to process variants B and C, the RI_app_ for process variant D decreases significantly at a post-heating temperature of 38 °C, followed by a further significant decrease when the post-heating temperature is increased to 45 °C.

Despite the different upstream and downstream thermal inputs for all process variants, post-heating has the same directional effect on the RI_app_. Two details are noted in this respect. First, for process variants with downstream pre-concentration heating (process variants A, B and C), the RI_app_ decreases to a lesser degree with increases in post-heating temperature compared to the process variant without downstream pre-concentration heating (process variant D). Second, the lowest RI_app_ values are approximately the same for all process variants and achieved at the same post-heating temperature (at 54 °C and range between RI_app_ 1.38–1.40) (Table 2).

There are a number of changes to rheological characteristics when each process variant is subjected to post-heating, as listed in Table 2. For example, post-heating of process variant A at 45 °C results in a significant reduction of $\delta_{10\;rad/s}$ and an increase of ΔE compared to without post-heating (Table 2). For process variant A, an increase in post-heating temperature to 54 °C results in a significant increase of ${G^{\prime }}_{10\;rad/s}$, *σ*_y_, and $\eta_{100\;1/s}$, and a further significant increase of ΔE (Table 2). Already at a post-heating temperature of 38 °C, the ΔE of process variant B increases significantly compared to without post-heating and at 23 °C. Additionally, for process variant B, the $\eta_{100\;1/s}$ increases significantly at a post-heating temperature of 45 °C compared to without post-heating (Table 2). Furthermore, the $\delta_{10\;rad/s}$ decreases significantly at a post-heating temperature of 45 °C compared to without post-heating at 23 and 38 °C. At a post-heating temperature of 54 °C, the ${G^{\prime }}_{10\;rad/s}$ for process variant B increases significantly compared to all other post-heating temperatures. Both the $\eta_{100\;1/s}$ and ΔE for process variant B increase significantly when the post-heating temperature increases from 45 to 54 °C (Table 2).

Compared to process variants A and B, more rheological characteristics listed in Table 2 change at lower temperatures for process variant C. For example, already at a post-heating temperature of 38 °C, there is a significant increase of ${G^{\prime }}_{10\;rad/s}$, *σ*_y_, $\eta_{100\;1/s}$, and ΔE for process variant C. With an increase in post-heating temperature to 45 °C for process variant C, there is a further significant increase of *σ*_y_, $\eta_{100\;1/s}$, and ΔE (Table 2). Finally, there is an additional significant increase of ${G^{\prime }}_{10\;rad/s}$, *σ*_y_, $\eta_{100\;1/s}$, and ΔE with an increase in post-heating temperature from 45 to 54 °C (Table 2).

Similar to process variant C, several rheological characteristics of process variant D increase significantly at temperatures greater than or equal to 38 °C (Table 2). For example, the *σ*_y_ at 38 °C is significantly greater than at 23 °C and without post-heating for process variant D. Furthermore, the ${G^{\prime }}_{10\;rad/s}$ and ΔE for process variant D with post-heating at 38 °C are significantly higher than for without post-heating, though not significantly higher than for post-heating at 23 °C (Table 2). With an increase from 38 to 45 °C post-heating temperature, there is a significant increase of the *σ*_y_, $\eta_{100\;1/s}$, and ΔE, analogous to the changes observed for process variant C at these temperatures. Unlike process variants A, B and C, there are no further significant increases in rheological characteristics of process variant D with an increase in post-heating temperature from 45 to 54 °C (Table 2).

Compared to samples without post-heating, for fat-free fermented concentrated MPb microgel dispersions post-heated at 38 °C for 300 min, the omission of pre-concentration heating was associated with a significant (post-heating) increase in particle size and voluminosity, whereas inclusion of pre-concentration heating was associated with significant increases in voluminosity and no change in particle size [23]. The present study confirms this conclusion. In addition, the extent to which the post-heating temperature affects rheological properties increases with upstream heat treatment intensity (combined with pre-concentration heating), where post-heating of process variant C (upstream: 95 °C for 256 s) has the largest factor changes in rheological characteristics compared to samples without post-heating (Table 2 & Figure 1). A slight difference is found when comparing the post-heating results for process variants C and D, where the values with increasing post-heating temperatures change to a lesser extent for process variant C (Table 2). It is postulated that the inclusion of pre-concentration heating reduces the potential for increasing voluminosity by way of post-heating, resulting in smaller changes in rheological characteristics for higher post-heating temperatures.

### 3.4. Summary and Application

The results of the present study provide insight into how upstream, downstream and post-production thermal inputs affect the microgel particles and, thereby, the texture of fat-free fermented concentrated milk products. From the conclusions drawn in Section 3.1, Section 3.2, Section 3.3, qualitative relationships between thermal inputs and evaluated textural parameters were defined and are depicted in Table 3.

While not affecting particle size, decreasing upstream thermal input in combination with pre-concentration heating (Figure 1 & Table 3: process variants C → B → A) leads to decreased gel firmness and apparent viscosity (at constant protein contents). While protein was a controlled variable in this study, further examinations regarding the effects on protein content should be carried out, based on increased involvement of whey proteins in the gel structure with higher upstream thermal input [16]. Omitting the pre-concentration heating step leads to smaller particles and weaker yielding behavior, with a limited effect on gel firmness (Figure 1 & Table 3: process variants C → D). While for process variants with and without pre-concentration heating, post-heating always leads to increases in rheological properties (e.g., gel firmness and apparent viscosity), significant post-heating induced particle aggregation is only seen for the process variant without pre-concentration heating (Table 3: process variant D; 6 → 54 °C) and for the highest temperatures of post-heating for process variants with pre-concentration heating (Table 3: process variants A, B, and C; 6 → 54 °C). Further investigations should be carried out regarding sensory characteristics of the final dispersions produced using these process parameters: The accompanying increases in particle size may be associated with graininess [22], whereas particles with different characteristics (e.g., firmness) may be perceived differently, e.g., gritty and hard [38].

Based on these results, it is postulated that combining different levels of thermal inputs upstream (milk), downstream (pre-concentration) and post-production, can produce fat-free fermented concentrated MPb microgel dispersions with similar textures. Furthermore, textural changes caused by altered thermal input at one point in the production scheme (e.g., lower upstream thermal input) can be rectified by thermal input at a later stage (e.g., post-heating).

## 4. Conclusions

This study showed that different combinations of thermal input at different points in the production scheme for fat-free fermented concentrated milk products, such as yoghurt and fresh cheese, are capable of producing similar and different textures. Depending on the point in the production scheme where thermal input is modified (upstream heating of milk *versus* downstream pre-concentration heating *versus* post-heating of the final product), various textural changes occur. Theses textural changes, brought about by different combinations of thermal input, are due to changes in particle size and voluminosity. Increasing the intensity of milk heating, including pre-concentration heating, and increasing post-heating temperatures result in increased gel firmness and apparent viscosity. Omitting downstream pre-concentration heating leads to smaller particles than when this heating step is included. However, the particles in these fat-free fermented concentrated MPb microgel dispersions are susceptible to heat-induced aggregation. Combined with pre-concentration heating, the intensity of upstream heating of milk does not significantly affect particle size or post-heating particle aggregation potential (only at very high post-heating temperatures). The results of this study demonstrate the possibility of apply defined thermal input combinations at different points in the production scheme to achieve specific textures of fat-free fermented concentrated MPb microgel dispersions. The impacts of these thermal input combinations on sensory characteristics and syneresis should be explored in future studies.

## Figures and Tables

**Table 1:** Processing conditions, gross composition and physical-chemical characteristics of fat-free fermented concentrated milk protein-based microgel dispersions process variants A, B, C and D at different stages during production (i ≥ 3; *n* ≥ 2; mean ± standard error).

		Process Variant			
		A	B	C	D
	i	3	3	4	5
Milk	pH_20 °C_	6.72 ± 0.02 ^a^	6.68 ± 0.04 ^a^	6.64 ± 0.02 ^a^	6.67 ± 0.04 ^a^
	Protein% *w*/*w*	3.4 ± 0.2 ^a^	3.7 ± 0.2 ^a^	3.6 ± 0.2 ^a^	3.5 ± 0.2 ^a^
Fermented milk gel	pH_20 °C_	4.52 ± 0.04 ^a^	4.49 ± 0.02 ^a^	4.49 ± 0.01 ^a^	4.49 ± 0.03 ^a^
Permeate	pH_20 °C_	4.52 ± 0.01 ^a^	4.47 ± 0.01 ^a^	4.48 ± 0.01 ^a^	4.48 ± 0.02 ^a^
	Protein% *w*/*w*	1.0 ± 0.2 ^c^	0.9 ± 0.2 ^bc^	0.6 ± 0.2 ^ab^	0.6 ± 0.1 ^a^
Final fresh cheese	pH_20 °C_	4.55 ± 0.01 ^a^	4.50 ± 0.01 ^a^	4.51 ± 0.02 ^a^	4.51 ± 0.02 ^a^
	Protein% *w*/*w*	8.6 ± 0.4 ^a^	8.8 ± 0.5 ^a^	9.1 ± 0.8 ^a^	8.8 ± 0.3 ^a^
	Dry matter% *w*/*w*	14.0 ± 0.4 ^a^	14.0 ± 0.4 ^a^	14.4 ± 0.5 ^a^	14.2 ± 0.3 ^a^

Lowercase letters signify significance within each row (*p* < 0.05).

**Table 2:** Mean values of optical, particle size and rheological properties of fat-free fermented concentrated milk protein-based microgel dispersions process variants A, B, C and D (8.2–9.9% *w*/*w* protein) produced with different heat treatments upstream, downstream (pre-concentration) and post-production (post-heating) (i ≥ 3; *n* = 2).

		**Particle Size**			**Rheology**				
**Post-Heating Temperature**					Oscillatory			Rotational	
				Span	Storage Modulus	Loss Tangent	Yield Stress	Apparent Viscosity	Energy Loss
	RI_app_ -	d_50,3_ µm	d_75,3_ µm	d_90,3_–d_10,3_ µm	${G^{\prime }}_{10\;rad/s}$ Pa	$\delta_{10\;rad/s}$ -	$\sigma_{y}$ Pa	$\eta_{100\;1/s}$ Pa s	ΔE J/m^3^
**Variant A**									
**Without** **post-heating**	**1.40 ^b,A^**	**11.3 ^a,B^**	**19.2 ^a,B^**	**30.0 ^a,B^**	**26 ^a,A^**	**23.5 ^b,B^**	**0.6 ^a,A^**	**0.5 ^a,A^**	**63 ^a,A^**
**23 °C**	1.40 ^b^	11.5 ^a^	21.3 ^ab^	32.7 ^a^	16 ^a^	26.3 ^c^	0.2 ^a^	0.5 ^a^	61 ^a^
**38 °C**	1.40 ^b^	11.3 ^a^	19.8 ^a^	29.3 ^a^	12 ^a^	28.3 ^c^	0.2 ^a^	0.6 ^a^	76 ^a^
**45 °C**	1.39 ^a^	12.0 ^b^	20.8 ^a^	30.9 ^a^	75 ^a^	18.2 ^a^	1.7 ^a^	0.7 ^ab^	100 ^b^
**54 °C**	1.39 ^a^	13.1 ^c^	23.2 ^b^	37.1 ^a^	522 ^b^	16.0 ^a^	8.7 ^b^	0.9 ^b^	143 ^c^
**Variant B**									
**Without** **post-heating**	**1.45 ^b,B^**	**11.4 ^a,B^**	**20.0 ^a,B^**	**25.9 ^a,B^**	**60 ^a,A^**	**20.1 ^b.B^**	**1.1 ^a,A^**	**0.7 ^a,A^**	**101 ^a,A^**
**23 °C**	1.47 ^b^	11.2 ^a^	19.9 ^a^	26.7 ^a^	66 ^a^	20.2 ^b^	1.1 ^a^	0.8 ^ab^	120 ^a^
**38 °C**	1.42 ^a^	10.9 ^a^	19.1 ^a^	25.4 ^a^	95 ^a^	19.1 ^b^	3.8 ^a^	0.8 ^ab^	128 ^b^
**45 °C**	1.43 ^a^	11.3 ^a^	19.0 ^a^	24.2 ^a^	437 ^a^	15.7 ^a^	14.6 ^ab^	1.0 ^b^	184 ^c^
**54 °C**	1.40 ^a^	13.0 ^a^	20.6 ^a^	25.8 ^a^	1227 ^b^	15.5 ^a^	28.5 ^b^	1.2 ^c^	252 ^d^
**Variant C**									
**Without** **post-heating**	**1.46 ^b,B^**	**11.9 ^a,B^**	**19.6 ^a,B^**	**26.5 ^a,B^**	**562 ^a,B^**	**15.5 ^a,A^**	**7.1 ^a,C^**	**1.3 ^a,B^**	**185 ^a,B^**
**23 °C**	1.47 ^b^	11.5 ^a^	19.5 ^a^	26.3 ^a^	531 ^a^	15.9 ^a^	10.4 ^a^	1.1 ^a^	159 ^a^
**38 °C**	1.40 ^a^	12.1 ^a^	18.6 ^a^	24.6 ^a^	1816 ^b^	14.6 ^a^	35.4 ^b^	1.5 ^b^	261 ^b^
**45 °C**	1.39 ^a^	12.9 ^b^	19.7 ^a^	23.9 ^a^	2183 ^b^	15.7 ^a^	49.6 ^c^	1.8 ^c^	295 ^c^
**54 °C**	1.38 ^a^	17.9 ^c^	28.7 ^b^	35.7 ^b^	2931 ^c^	15.0 ^a^	71.1 ^d^	2.0 ^d^	368 ^d^
**Variant D**									
**Without** **post-heating**	**1.76 ^c,C^**	**4.2 ^a,A^**	**5.7 ^a,A^**	**4.9 ^a,A^**	**513 ^a,B^**	**16.8 ^b,A^**	**3.9 ^a,B^**	**1.0 ^a,B^**	**107 ^a,A^**
**23 °C**	1.77 ^c^	4.3 ^a^	6.1 ^a^	6.3 ^a^	785 ^ab^	14.7 ^a^	7.7 ^a^	1.2 ^a^	174 ^ab^
**38 °C**	1.60 ^b^	10.0 ^b^	17.3 ^b^	26.2 ^b^	1444 ^bc^	14.5 ^a^	27.2 ^b^	1.5 ^a^	257 ^b^
**45 °C**	1.43 ^a^	17.0 ^c^	28.9 ^c^	43.7 ^c^	2306 ^cd^	14.3 ^a^	55.9 ^c^	2.2 ^b^	371 ^c^
**54 °C**	1.38 ^a^	32.4 ^d^	66.9 ^d^	116.0 ^d^	2501 ^d^	14.8 ^a^	72.7 ^c^	2.3 ^b^	396 ^c^

Lowercase letters represent differences between post-heating temperatures of the same process variant; uppercase letters represent differences between the processing variants without post-treatment (6 °C); significance using *p* < 0.05 determined using ANCOVA with protein as a covariate. Protein was nested within post-heating temperature.

**Table 3:** Qualitative overview: Relative impacts of combinations of upstream (milk heating), downstream (pre-concentration heating) and post-heating thermal inputs for process variants A, B, C and D on particle and rheological properties.

		Upstream Milk Heating		Downstream Pre-Concentration Heating	
				(With → Without)	(Without)
Parameter		Without Post-Heating	With Post-Heating	Without Post-Heating	With Post-Heating
		Process Variants C → B → A	Process Variants A, B, and C 6 → 54 °C	Process Variants C → D	Process Variant D 6 → 54 °C
Apparent refractive index	RI_app_	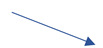	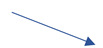	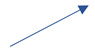	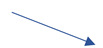
Particle size	d_50,3_	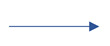	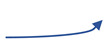	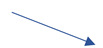	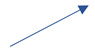
	d_75,3_	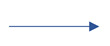	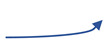	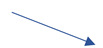	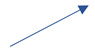
Span	d_90,3–_d_10,3_	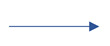	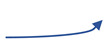	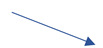	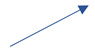
Storage modulus	${G^{\prime }}_{10\;rad/s}$	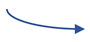	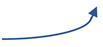	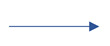	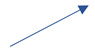
Loss tangent	$\delta_{10\;rad/s}$	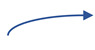	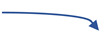	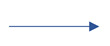	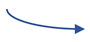
Yield stress	$\sigma_{y}$	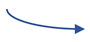	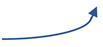	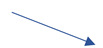	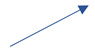
Apparent viscosity	$\eta_{100\;1/s}\;$	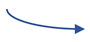	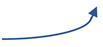	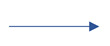	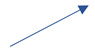
Energy loss	ΔE	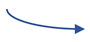	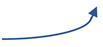	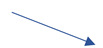	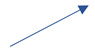

Arrows in this table indicate relative qualitative directional effects for each parameter (left column) in relation to the process variants or post-heating temperature (top rows, indicated in blue): The following are some example of how to read the arrows: An arrow decreasing linearly from left to right indicates that for this set of process variants or post-heating temperatures, this parameter decreases generally in a linear fashion; a horizontal arrow indicates no change for this parameter; and an arrow increasing sharply followed by smaller increase indicates that (for the set of process variants or post-heating temperatures), this parameter increases largely initially, followed by a smaller increase.

## Data Availability

Not applicable.
